# Need for Sustainable Packaging: An Overview

**DOI:** 10.3390/polym14204430

**Published:** 2022-10-20

**Authors:** Idowu David Ibrahim, Yskandar Hamam, Emmanuel Rotimi Sadiku, Julius Musyoka Ndambuki, Williams Kehinde Kupolati, Tamba Jamiru, Azunna Agwo Eze, Jacques Snyman

**Affiliations:** 1 Institute for NanoEngineering Research (INER) and Department of Chemical, Metallurgy and Materials Engineering, Tshwane University of Technology Pretoria South Africa, Pretoria 0183, South Africa; 2 École Supérieure d'Ingénieurs en Électrotechnique et Électronique, 93162 Paris, France; 3 Department of Electrical Engineering, Tshwane University of Technology Pretoria South Africa, Pretoria 0183, South Africa; 4 Department of Civil Engineering, Tshwane University of Technology Pretoria South Africa, Pretoria 0183, South Africa; 5 Department of Mechanical Engineering, Tshwane University of Technology Pretoria South Africa, Pretoria 0183, South Africa

**Keywords:** sustainability, biodegradable, biopolymer, pharmaceutical products

## Abstract

Packaging materials are a significant part of our lives due to their daily usage at grocery stores, supermarkets, restaurants, pharmaceuticals, etc. Packaging plays an important role in ensuring that the products are preserved during handling, transporting, and storage. Similarly, it helps to maintain and prolong the shelf life of products. These materials are used for packaging meats, poultry and seafood products, food and beverages, cosmetics, and pharmaceutical products. Several applications of packaging materials have been discussed extensively, with little discussion on their end of life and continuous availability without impacting the environment. This study presents the need for sustainable packaging as a result of growing demands and the environmental impact of packaging materials after use. This study also presents the importance, types, and applications of packaging materials. Based on the findings of this study, sustainable packaging is made possible by using bio-based and recyclable materials. These materials contribute a great deal to protecting and ensuring a sustainable environment.

## 1. Introduction

Sustainability is mostly categorized into three major dimensions, namely, human well-being, the economy, and the environment [1]. These three categories can be regarded as a means of improving human well-being (i.e., fair burden-sharing and social equity) and maintaining the resilience of the ecosystem [2]. Looking at sustainability from the perspective of ecology, there is the need for contribution to ensure that the environment and a healthy ecosystem are maintained at all times. This can also be linked to packaging materials. Packaging materials are said to be sustainable if the usage of virgin resources is reduced and if post-consumed materials are recyclable or reusable from readily available materials [3]. Material sustainability is a function of several factors ranging from the economic to the environmental, including costs and impacts, the functionality of aesthetic properties, the production to the processing of end-of-life, and from local- to global-scale effects [3]. According to some authors, achieving absolute sustainability is a major challenge, if not an almost impossible one [4,5].

In a survey conducted by de Feo et al., it was reported that in terms of environmental sustainability, glass bottles are more sustainable than aluminum cans and plastic bottles [6]. The authors further explained that the reason for this conclusion was based on the general/unscientific belief and/or incorrect communication by the scientific community to the citizens.

Globally, the largest market usage of plastic is for packaging, resulting in almost half of the plastic waste produced [7]. As explained by Defruyt, only 2% of plastic packaging materials are recycled as packaging materials globally, while the remaining are either incinerated or end up in landfills, water bodies, and the environment. Furthermore, sustainability is more than simply improving material collection and recycling but taking a closer look at what kinds of materials are in the market. As highlighted by the author, 30% of plastic-based packaging items are either too complex or too small to recycle; examples include multi-layered materials and small sachets/wrappers, respectively [8]. Despite these challenges, packaging materials will always be a part of our lives.

Packaging materials are a significant part of our lives due to their daily usage at grocery stores, supermarkets, restaurants, pharmaceuticals, etc. Packaging plays an important role in ensuring that products or contents are preserved during handling, transporting and storage. It also helps to maintain and prolong the shelf life of the products. Hamouda [9] explained that packaging plays an important role during transportation and storage, as well as preventing contamination and preserving the product’s freshness. Similarly, Schmid and Agulla [10] explained that food packaging materials ensure that the foodstuffs are adequately protected against external influences or damages. Packaging is also used to instruction and instructions about the product and sometimes for promotional sales [11].

Packaging materials contribute to the cost of the product due to the materials used and the process of producing them. The environmental influence of such materials, either during production or after their end-of-life, is of major concern, leading to several kinds of research along that line. Therefore, there is a need to critically evaluate the various materials used for packaging; the areas of application for each material and the merits and demerits of each packaging material are equally important. Several materials used as packaging materials include but are not limited to plastic, paper, glass, metal, etc. The nature of the product to be packaged will determine the choice of packaging materials. Environmental concern is another influencing factor when it comes to the choice of material selection. Of all the packaging materials, plastic materials seem to be the most widely used material due to their various merits, such as their lightweight qualities, low cost, moldability, flowable nature, variable colors, or transparency [12]. To ensure the availability of packaging materials all year round without negatively affecting the environment, a sustainable approach needs to be investigated. These approaches could be material recycling and reuse [13,14], the choice of materials [15], and the use of bio-based and biodegradable materials [16,17]. Thus, the study focuses on the need for sustainable packaging.

## 2. Types of Packaging Materials

Packaging materials play a significant role when it comes to the quality of the product. In a case where the packaging material is used to provide information, the material must be such that it can accommodate the printed text or images. In this section, we looked at various packaging materials and highlighted the sources and methods of production. The link between the customers and the manufacturer is the quality of the packaging material, which guarantees the confidence the customers have in the product [18]. The selection of materials used for packaging is an important aspect of product presentation and preservation. The type of product is equally a determining factor when it comes to the choice of packaging materials. Various industries (food, cosmetics, pharmaceuticals, meat, etc.) have specific materials that are best suited to their products and services. Hence, there is a need for the discussion of different materials in detail.

### 2.1. Plastics

Petroleum-based polymeric materials have been widely used as packaging materials. Most of these polymers are polyethylene (PE), polypropylene (PP), polystyrene (PS) and polyester (PET) [18]. Plastic-based materials are the most used packaging materials, and about 26% of the total usage of polymers in packaging makes it the largest application of plastic materials [19]. The usage of plastics is expected to double within the next duration of 20 years since it is fast replacing other materials used for packaging. This is due to inherent characteristics, such as good barrier properties, a lightweight quality, low cost, etc. [20,21].

With all the numerous benefits of plastic packaging materials comes its downside due to its negative environmental impact. The production of petroleum-based materials comes with the release of carbon dioxide (CO_2_) into the atmosphere. Furthermore, the improper handling of packaging plastics or collection/recycling will make them end up in landfills and water bodies, therefore, polluting and contaminating the land and the oceans [22,23]. Many industries are looking into alternative sustainable and environmentally friendly materials. This is also a result of the European Union (EU) Commission’s goal to reduce plastic waste by 55% in 2025 and ensure that materials are100% recyclable or reusable by 2030 [24]. The recycling of plastic materials with several layers of different materials is challenging and not cost-effective. Hence, there is an urgent need for sustainable and environmentally friendly materials to overcome these challenges. Several researchers are focusing on biodegradable materials with keen attention to enhancing the physical and mechanical properties of bio-based packaging materials [25,26].

**Figure 1:**
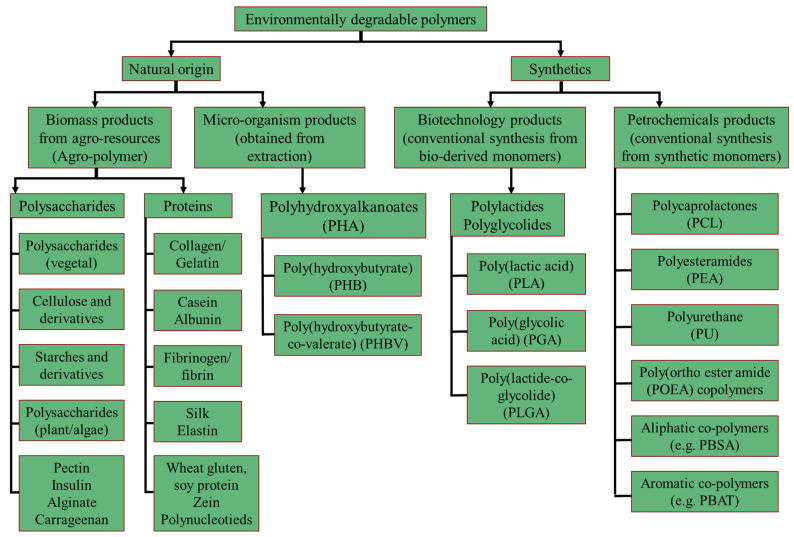
Classifications of biopolymer and biodegradable materials. Adopted from [30].

Biodegradable polymers can either be of natural or synthetic origin. There is a difference between biopolymer materials and biodegradable materials. All biopolymer materials are biodegradable, but not all biodegradable materials are biopolymers [27]. Biopolymers can be made from renewable resources, e.g., starch, while biodegradable materials are materials that can decompose into inorganic compounds, such as carbon dioxide, methane, water, or biomass [9]. Figure 1 shows the various classifications of biopolymer and biodegradable materials. The main three sources of biopolymer materials are as follows [28,29]:

- Biomass sources: the extraction of biopolymers directly from polysaccharides (e.g., cellulose, starch, galactomannans) and proteins (e.g., gluten and casein)
- Microorganism sources: the production of biopolymers by microorganisms. These polymers include polyhydroxyalkanoates (PHA) and polysaccharides.
- Chemical sources: the chemical synthesizing of bio-based monomers, such as polylactic-acid (PLA), and the lactic acid-based thermoplastic aliphatic polyester.

#### Drawbacks of Plastic Packaging Materials

**Figure 2:**
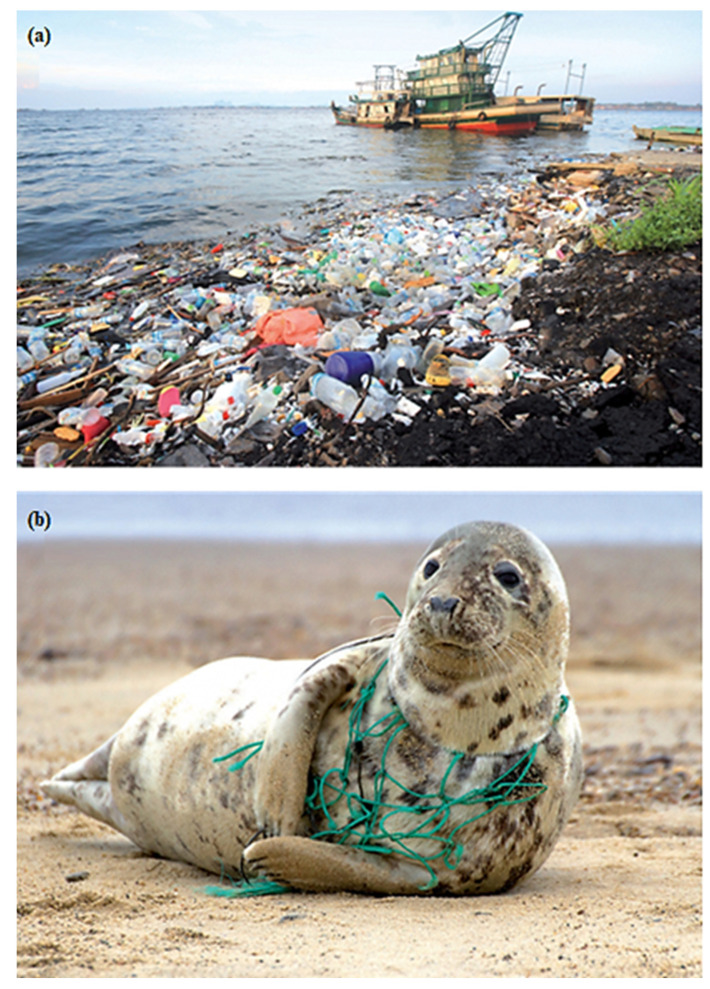
The catastrophic impact of plastic materials on (**a**) water bodies and (**b**) aquatic animals. Adopted from [22].

The huge and increasing use of plastic materials for packaging has witnessed tremendous growth in the past few decades due to low costs and easy processability. However, with the good sides come the bad in terms of the health and environmental hazards that come with the production, usage, and disposal of plastics used as packaging. The inappropriate disposal and recycling mechanism can lead to an accumulation in landfills, which gradually find their way downstream into water bodies, and ultimately into the ocean, causing serious havoc, as shown in Figure 2. Most plastic materials used for packaging have a low melting point, and therefore, they are not useful for high-heat applications. Furthermore, the slow rate of biodegradation experienced by such materials is another drawback associated with plastic-based packaging materials [31].

### 2.2. Paper and Paperboard

The use of paper and paperboard for packaging has been widely seen in the food and beverages industries, furniture, tobacco, building and construction materials, machinery, electrical and electronics equipment, etc. [32]. Hardly will you find a product in the market that is unpacked. The reason for this packaging is for safety, easy handling, product protection, providing information to consumers, etc. Paper and paperboard have superior advantages compared to plastics, metals, and glass as packaging materials in terms of their sustainability and cost. However, this material has some limitations, such as an inferior resistance to water, chemical and low strength [33]. Paper is next to plastic in terms of materials used for packaging [34]. The reason why plastic-based packaging materials are gaining so much ground in the packaging market despite their hugely negative environmental impacts is due to their strength, permeability, stability, easy-to-sterilize nature, transparency, and high liquid resistant properties [35]. The environmental issues surrounding the use of plastic-based packaging give rise to paper and paperboards as packaging materials.

**Figure 3:**
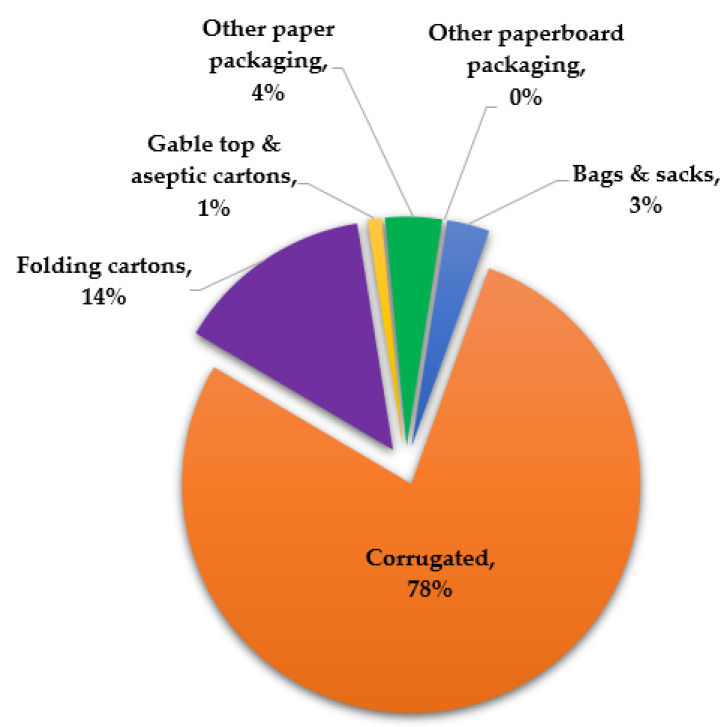
Shares of paper/paperboard as packaging materials in the USA. Adopted from [40].

Paper and paperboard are sheet materials that consist of interlaced cellulose fiber networks that are extremely susceptible to moisture or liquid due to the hydrophilic nature of cellulose fibers [33]. To overcome this, materials with better moisture and water barriers, such as plastic and aluminum, are incorporated [36,37]. Coles [38] and Riley [39] explained that treatments such as laminations and coatings could extend the applications of packaging materials and equally extend the shelf life of the products placed inside them. Paper and paperboard can be used in various forms, as shown in Figure 3.

Paper can either be used directly or as a passive packaging material. Paper packaging materials could be used as cardboard surfaces on which to provide information, surrounding the product or product container, plate, carton, tray, etc. [41]. Packaging a liquid product with a paperboard requires some modifications to the material. Paper-based packaging is susceptible to swelling due to the absorption of moisture from the product. This is common with wet or moisture-related products. To avoid such occurrences, the paperboard can be coated with certain polymers, which provide the required barrier against chemical attacks and moisture absorption. The coating also provides some form of improvement to the paperboard, such as strengthening, chemical resistance, etc. The coating of polymers includes but are not limited to low-density polyethylene (LDPE), high-density polyethylene (HDPE), polypropylene (PP), and polyester (PET) [41]. The method of paperboard coating is either extrusion or roll coating. Packaged foods are sometimes prepared or reheated in a microwave oven without removing the packaging materials. The packaging materials are often coated based on the reasons mentioned above. The coating materials must be such that they will not react while in the microwave or oven, meaning they will not be heated by the microwaves [42]. If coating materials are reactive due to the microwave, it could lead to certain unwanted chemical reactions and/or contamination which could be harmful to humans. With the improvement, discoveries made and growing environmental concerns, paper-based packaging materials are fast gaining more attention and becoming a better form of packaging globally with improved performance.

### 2.3. Glass

**Figure 4:**
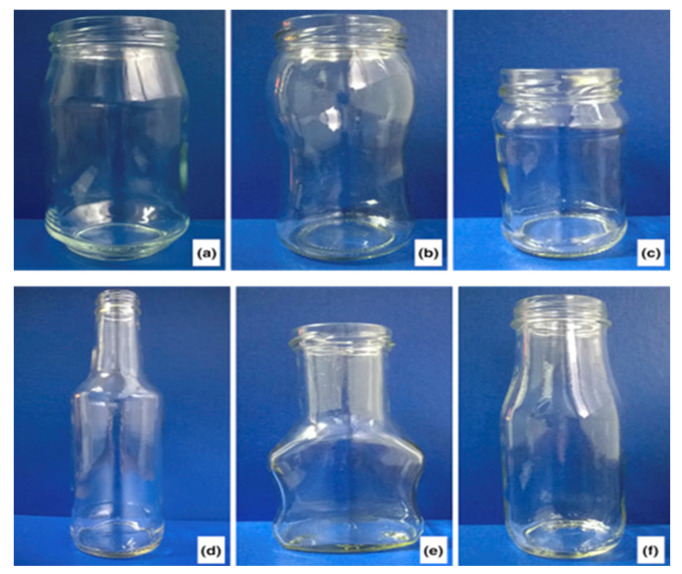
Various glass bottle shapes (**a**) wide mouth, (**b**) wide neck, (**c**) mini wide mouth, (**d**) longneck, (**e**) white clear glass and (**f**) milk glass jar.

Globally, glass has been recognized as an important material. The food and pharmaceutical industries still make use of glass for packaging, unlike other industries that have replaced it with other materials, such as plastics and metals. Several factors are responsible for the continuous use of glass for packaging in the food industry. These reasons include food safety and preservation, quality maintenance, sensorial attribute preservation, and resistance to chemical attacks [43]. Glass containers could be made from recycling used glass containers or by other methods which involve heating a mixture of silica, sodium carbonate, and limestone/calcium carbonate to an elevated temperature to melt the materials forming a thick liquid that is dispensed into a mold [44]. The glass containers can be molded into several shapes and sizes, as shown in Figure 4. The shapes, sizes and colors of glass bottles are sometimes used to communicate a message to the consumers by the product manufacturers. From the literature, it has been reported that packaging has a way of influencing the consumers, and therefore, product manufacturers pay keen attention to the product’s packaging material and design [45,46].

As explained by Franco and Falqué [47], ideal packaging materials must be resistant to hazards, be inert, and prevent molecular transfer, either to or from the packaged products. Glass bottles have been widely and satisfactorily used for packaging beverages and water, and certain dry products. Glass containers have a significant yet declining role in packaging industries, especially in the food and beverage industries [48]. Although there is a decreasing usage of glass-based packaging materials, they will always remain one of the safest packaging materials in the food and beverage industry [49].

#### Drawbacks of Glass Packaging Materials

Just like every other packaging material, glass packaging has its limitations, such as a high weight, breakage, and fragility to thermal expansion or contraction. Glass packaging is not ideal for extreme temperatures. When glass is not properly handled during handling and transit, it can lead to physical hazards [43]. The temperature of glass packaging should not exceed 65 °C and the cooling of glass containers should not rapidly exceed a 40 °C differential [50].

### 2.4. Metal

**Figure 5:**
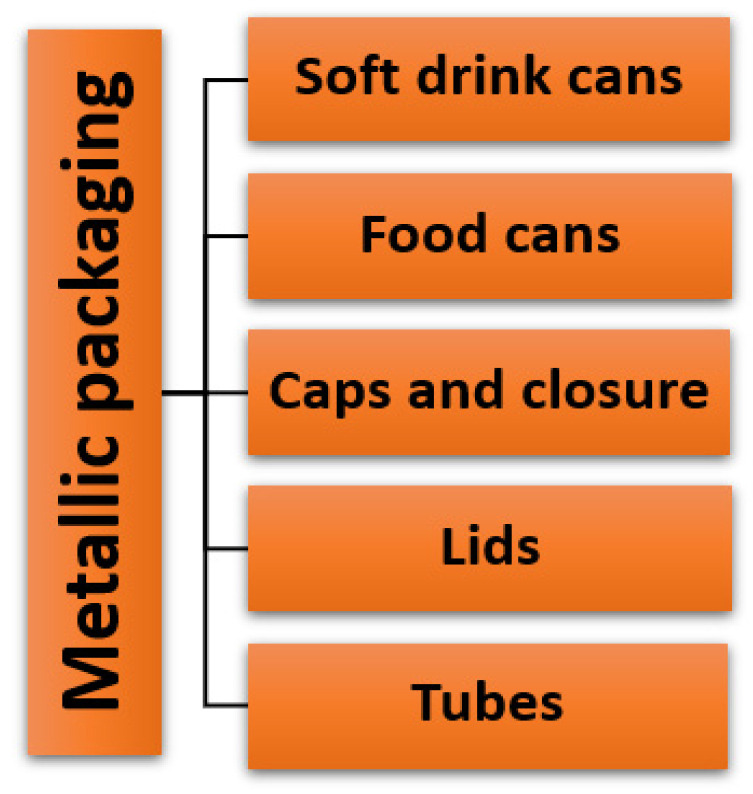
Uses of metals and metalloids as packaging materials.

The packaging materials and its content most of the time have direct contact, especially in the case of can drinks. The health and safety of the consumers are paramount to the manufacturers when it comes to metal-based packaging materials [51]. Therefore, they must comply with the basic regulation and carry out a risk assessment regularly to ensure that there is no harmful interaction between the content and the container. Several metals are widely used for packing. These include aluminum, tin, lead, chromium, etc. Among all these metals, aluminum is the most used material for packaging due to its inherent properties, such as having a low cost, being lightweight, flexible, recyclable, having a high heat resistance, etc. Figure 5 shows the possible applications of metals as packaging materials. For the sake of this study, only the above-mentioned metals are discussed.

- Aluminum (Al)–It has excellent properties, such as recyclability, decorative potential, formability, physical protection, etc., making it the most used metal-based material for packaging [41]. Packaging materials are not made from pure aluminum; instead, alloys of aluminum are made by alloying it with other alloying metals, such as zinc, copper, silver, iron, and manganese. Traces of these metals can often be found in the content of the container when they corrode [52]. A high intake of Aℓ may lead to certain disorders in humans, and therefore, the content of corroded containers should not be consumed. Aluminum is used for canned drinks, and it can also be in the form of foil. The foil can either be thin or thick. Thin foils are often used to wrap food, while thick foils are used for trays, baking pans, etc. Foil provides a brilliant barrier to moisture, light temperatures, air odors and microorganisms. The use of aluminum-based packaging materials in microwave heating has been a major concern. Walsh and Kerry [41] reported in their study how the Fraunhofer Institute for Process Engineering and Packaging IVV in Freising, Germany, concluded that aluminum-based packaged food could be heated in a microwave. This was based on over 200 food samples that were heated in the microwave without any harmful outcomes recorded. Aluminum-based materials also give more uniformity in heating than other viable materials. The only drawback is that it takes about three times the normal time to heat in a microwave. Thus, this makes it an unpopular choice when it comes to the microwave heating of aluminum-based packaging materials [53].
- Tin (Sn)–This is often used to coat steel when used for packaging foodstuffs, and this is often achieved by the electrochemical deposition of tin onto the surface of the metal being coated. The area of application determines the level of coating, which ranges from 2.8 to 15.4 g/m^2^ [51]. Tin-coated containers should not be used directly for wet content due to the possibility of it dissolving in its content. This is why tin-coated metals used for wet food content are usually shielded with an organic protective coating. For dry foods, the tin-coated container may not be protected with an organic protective coating. To prevent any extra consumption of tin, or other metallic substances, there is a regulation on acceptable limits. The EU regulation limits the levels of tin in canned foods (excluding beverages) and beverages and foodstuffs for infants, respectively, to 200, 100 and 50 mg/kg. In most non-EU countries, the Codex Alimentarius advises limits of between 150 and 250 mg/kg for liquid and dry foods, respectively [51].
- Lead (Pb)–Lead-based containers are directly used for packaging, but they are also found in containers made from tin. This is possible due to Sn and Pb coexisting in the ore, and therefore, Pb contaminates the Sn and ultimately contaminates the food content if proper and due diligence is not followed in terms of the regulations surrounding the allowable lead limit in foodstuffs. According to EU regulation No 466/2001, the traces of lead content in foodstuffs should be within a range from 0.02 to 0.1 mg/kg. As explained in the European Standard EN 10333, the level of lead in tin-coated packaging containers has been reduced to a maximum of 100 mg/kg by industrial action in the USA and Europe [51]. Lead is toxic and could damage the organs in the human body, and the central nervous system could also be seriously damaged [54]. A high level of lead in children could cause convulsions, mental retardation, and encephalopathy (brain disease) [55].
- Chromium (Cr)–This is mostly used in tin-based containers, such as cans as a thin layer to improve their properties, including strength and stability against high oxidation levels [54]. High toxicity is associated with Cr and hexavalent forms, which could impact living organisms severely due to mutagenic and carcinogenic properties [56]. The level of Cr in tin-coated foodstuff containers is negligible and, thus, not of any health concern. There is no regulation for the Cr content in foodstuff, but for drinking water, the World Health Organization (WHO) has it limited to 0.025 mg/L.

## 3. Areas of Application of Packaging Materials

Packaging materials add many values to their products and protect the content from contamination. The product will determine what type of packaging material to use. Similarly, the cost of producing the packaging materials and the environmental concerns are other determining factors. There are several regulations on the choice of material selection for dry and wet packaging. The areas of application for sustainable packaging materials are discussed in the following subheadings.

### 3.1. Food

The shelf life of packaged food is a function of the quality of the packaging materials; therefore, adequate attention should be paid to the choice of material and the manufacturing process used in making the food packaging materials. One of the major areas where packaging materials are largely used is the food industry. The increasing growth of the global population and the advancement in technology is responsible for the high demand for packaged food. Various requirements need to be satisfied by packaged products, and these are economical, effective, and efficient concerns [57]. How the food is produced, distributed, and consumed reflects the need for continuous improvement in the packaging materials.

The continuous demands for healthier, more convenient, safe, and extended shelf lives are responsible for the growing innovative and new methods of food packaging. These materials are made from polymers, paper/paperboards, metals, or glass. In the case of polymers, they can either be edible or non-edible. Non-edibles are mostly made from petrochemicals, while edible films are made from renewable sources, including starch, carbohydrates, polysaccharides, etc. [48,49,50,51,52,53,54,55,56,57,58,59,60]. Edible packaging materials are known to be suitable for direct human consumption without any adverse effects on health. These materials can either be directly applied onto the food surfaces (edible coating) or formed separately and placed on the food as a thin film/sheet (edible film) [61].

**Figure 6:**
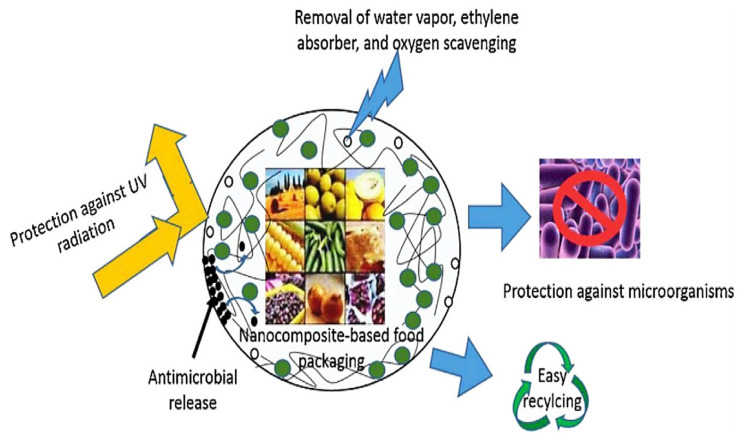
Importance of biodegradable and nanoparticles for food packaging materials. Adopted from [62].

In the case of polymers used to package food, they can either be biodegradable or non-biodegradable. The properties of these materials can also be improved by incorporating nanoparticles. Improved biodegradable-based packaging materials have several advantages over conventional (unimproved-non-biodegradable) packaging materials, as shown in Figure 6. Packaging materials will continue to find a useful application in the food and beverages industry due to the continuous demand, as mentioned previously, for safer, healthier, and the extended shelf lives of food products.

### 3.2. Cosmetics

Plastics and glass materials are the most widely used packaging materials in the cosmetics industry. This is due to the direct contact they have with the product. Paper/paperboard could also be used, but they would have to be coated with polymers or aluminum foil to prevent the packaging material from absorbing its content. Packaging plays an important role in protecting content against microbiological contamination and light and, in marketing, can provide information about the product [63]. Another important thing to consider when developing packaging materials for cosmetics, food, or pharmaceuticals, is leaching, which involves the migration of certain compounds from the packaging material to its content [64,65]. Thus, careful material selection and improvement should be the priority during the production of these materials. The desired properties have necessitated the development of biodegradable polymers for cosmetic packaging containers. The essence of this is the growing demand for new and improved materials and the afterlife of packaging materials. The environmental impacts of packaging materials are a serious concern because most packaging materials are not recyclable, and therefore, they end up in landfills or water bodies. Biodegradable polymers have the potential to degrade under specific environmental conditions and the action of certain living organisms [66]. These biodegradable polymers include poly (lactic acid), polysaccharides, PHAs, etc.

Another very important material used for the packaging of cosmetics is glass. Glass is known to be one of the oldest packaging materials. This is because glass is impermeable, nonporous, chemically inert, non-degradable, and recyclable [67]. Cosmetic glass containers come in different shapes and sizes. The glass containers can be used as perfume jars, lip balms, in eye shadow, liquid foundation, etc. It can also be clear or colored glass, depending on its content and attractiveness to customers. Irrespective of the properties possessed by other packaging materials, the option of glass will always be the choice material in the cosmetics industry.

### 3.3. Pharmaceuticals

Pharmaceutical products are chemical substances that can be synthetic or natural and have pharmacological or medical effects on the body [68]. These products are categorized based on their therapeutic effects, the way they are administered and their chemical properties [69]. Another important aspect to note, as explained by the author, is that they consist of antipyretics, analgesics, antibiotics, antiseptics, stimulants, antimalarials, stabilizers, statins, contraceptives, and tranquillizers. Furthermore, they have several targeting potentials, such as cardiovascular, digestive, central nervous, endocrine, respiratory, reproductive, urinary, and immune systems and organs (skin, musculoskeletal, ear, eye, and nose) [70]. Thus, pharmaceutical packaging plays an important role in ensuring that the product meets the required goals. This could be during production, transportation, storage, sales, delivery, and use [71]. Packaging materials protect pharmaceutical products from spoilage, loss of potency, contamination, unwanted environmental conditions (light, moisture, and oxygen) and provide information regarding the product and dosage [72].

**Figure 7:**
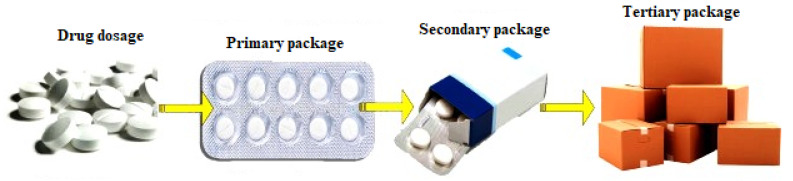
Packaging system for pharmaceutical products. Adapted from public domain [73].

Pharmaceutical products are largely packaged in plastics, paper, and glass. Pharmaceutical packaging is divided into three parts, namely: primary, secondary, and tertiary systems of packaging [70]. The primary system of packaging has direct contact with the medication, while the secondary system is the packaging outside of the primary container. The secondary packaging system could be a box, cardboard, or plastic crates. HVAX Viable Pharma Infrastructure described the tertiary system of packaging in the pharmaceutical industry as the package housing the secondary packaging system, as shown in Figure 7.

For a material to be considered a suitable pharmaceutical packaging material, it must meet the following criteria as presented by [72,74]:

- Adaptable to high-speed packaging machines
- Approved by the Food and Drug Administration (FDA)
- Does not impart odor or taste to the product
- Non-reactive with the product
- Non-toxic
- Prevention and preparedness against environmental conditions
- Protects the dosage form against breakage or damage
- Tamper-resistant when necessary.

### 3.4. Meat, Poultry and Seafood

A high water content in meat, poultry and seafood products can lead to the rapid growth of numerous microorganisms, pathogenic bacteria, and spoilage [75]. These products are also highly perishable within a very short time if they are not well preserved. Physical changes, such as discoloration (reduced redness, darkening of red meat, etc.), are the results of the improper packaging of meat, poultry, and seafood. Contamination is another hazard that is observable with these products during handling and processing, thereby leading to the reduced shelf life of the products and potential health challenges when consumed. Packaging materials can help to prevent contamination, loss of quality and also extend the shelf life of meat and poultry products. Packaging materials reduce the rate of microbial growth and limit reactions due to microbial enzymes. Winotapun et al. [76] explained that laminated polylactic acid (PLA) could enhance the odor barrier, leading to a reduced unwanted odor from meat, poultry, and seafood products.

Meat, poultry, and seafood product quality is best maintained at freezing temperatures, therefore extending shelf life. The combination of the right freezing temperature and the use of packaging with the right permeability further extends the shelf life and freshness of these products [77]. Higher storage temperatures and poor packaging often result in a loss of weight due to the sublimation of ice from the surface of frozen food products [78]. Similarly, packaging materials also make the product attractive to customers. Talking about shelf life, product shelf life is a function of three major factors, namely [79]:

- Environment–The physical condition to which the food products are exposed (e.g., light, relative humidity, temperature, and customer handling).
- Product characteristics–These include physical, chemical, biological, etc.
- Packaging–The property/quality of the packaging material.

The essence of packaging, in general, is in four categories, namely: (i) to protect the product from deterioration and loss of value, (ii) to communicate information regarding the product, (iii) convenience, and (iv) the containment of products of different sizes and shapes. Packaging materials for meat and poultry products can be made from edible and non-edible films. Edible films, such as starch and its derivatives (carrageenans, alginates, pectin, and cellulose ethers) extend shelf life by delaying the dehydration, surface browning, and oxidative rancidity of meat and poultry products [80].

## 4. Measures for Improving Sustainability in Packaging

**Figure 8:**
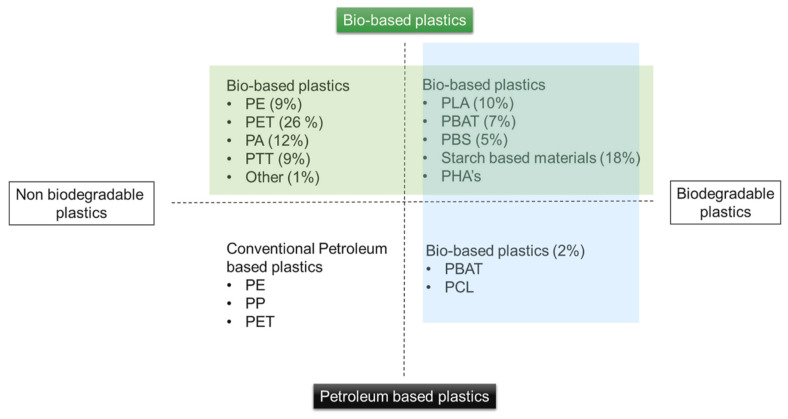
Relationship between bio-based plastics and the market share. Adopted from Mendes and Pedersen [19].

A high consumption of virgin materials could lead to the possible depletion of such materials. The scarcity of raw materials for the development of packaging materials is another possibility arising from the depletion of virgin materials. The exploitation of raw material (from their extraction to refined product) for packaging materials contributes to global environmental issues. To ensure sustainable packaging, there is a need to recycle used materials to create similar products or other possible products [81]. Packaging materials are of different types, ranging from paper to plastics and metals to glass. The sustainability of the packaging industry depends on several factors, such as the availability of raw materials, good recycling practices, the use of renewable resources and the effective and efficient policy on product packaging materials. Considering plastics, which is a type of packaging material, this product has been made for several decades from petroleum-based polymers such as polyethylene (PE), polypropylene (PP), polystyrene (PS) and polyester (PET) [82]. These petroleum-based polymers are non-biodegradable and possess a low recycling rate of less than 14% [58,83]. Therefore, to maintain sustainable plastic-based packaging materials, environmentally friendly biodegradable polymers need to be explored more than ever before. Biodegradable polymers such as proteins, polysaccharides, lipids, and vegetal sources (e.g., cellulose, starch, chitosan, corn zein, whey protein, waxes, collagen, etc.) have been widely researched [84,85]. Such materials should be encouraged for packaging materials. In addition, the government should develop and enforce existing policies on the use of biodegradable and 100% recyclable materials. Similarly, manufacturing companies for packaging materials should work towards achieving 100% recyclable materials. Figure 8 shows the relationship between biodegradable and non-biodegradable plastics when used as packaging materials. The market share of each bio-based plastic is shown in parenthesis, with PET having the highest market share, followed respectively by starch-based materials, PA, PLA, and so on.

Other packaging materials (such as glass, metals, and paper/paperboards) should have manufacturers base their production on protecting the environment—meaning they should consider materials that can be recycled and reused. Metals such as aluminum are 100% recyclable [86]. Recycled paper, when compared with virgin pulp, uses less water and energy while having a lesser negative impact on the environment [87]. Glass, on the other hand, can be reused or converted for other usages, such as fine aggregate in concrete or mortar [88,89,90,91,92]. The perfect way to measure the level of sustainability in packaging materials is the percentage recyclability of such material. Thus, manufacturers of packaging materials should work toward using biodegradable and sustainable materials that have a high recycling rate.

## 5. Conclusions

This study focused on the sustainability of packaging materials and the steps which should be undertaken to ensure and enhance sustainability. Packaging materials have shown significant importance for the protection and safety of their contents, and they have been widely used in various areas of life, such as meat, poultry and seafood, food and beverages, cosmetics, and pharmaceutical industries. Several packaging materials have found useful applications. These materials include plastics (petrochemical and biopolymer), paper and paperboard, glass, and metals. This study has presented the need for sustainable packaging, which is a result of the growing demands and environmental impact of packaging materials and material end-of-life. The study also presents the importance, types, and applications of packaging. Based on the findings of this study, the following drawbacks and solutions were discussed on how to ensure the sustainability of packaging materials:

- Plastics—an inappropriate disposal and recycling mechanism can lead to the accumulation of plastics in landfills and water bodies (causing serious havoc on the aquatic organisms). The best practices should be encouraged and enforced. Manufacturers should be restricted to using plastic materials that are 100% recyclable, biodegradable, reusable, etc.
- Paper—is prone to chemical attacks and moisture absorption. The paper or paperboard can be coated with certain polymers, which provide the required barrier against chemical attacks and moisture absorption. Furthermore, the coating provides some form of improvement, such as strength and chemical resistance.
- Glass—has limitations, such as a high weight, breakage, fragility to thermal expansions or contractions and is not ideal for extreme temperatures. Similarly, when not properly handled during handling and transit, it could lead to physical injuries. Glass is highly recyclable and can be used for other purposes, including aggregates in concrete.
- Metal—is prone to corrosion due to moisture; thus, it can be coated with certain metals. Careful consideration is encouraged to minimize metal interaction with its contents and ensure that metal contamination is within the allowable limit according to various global regulations.
- Problematic and/or unnecessary plastic packaging materials should be eradicated through innovative and environmentally friendly approaches.
- The general goal behind packaging materials should be to ensure that no material ends up as waste.

## Data Availability

Not applicable.
